# Optimization of Scanning and Counting Sensor Layout for Full Route Observability with a Bi-Level Programming Model

**DOI:** 10.3390/s18072286

**Published:** 2018-07-14

**Authors:** Donghui Shan, Xiaoduan Sun, Jianbei Liu, Ming Sun

**Affiliations:** 1 Traffic Safety and Digital Technology R&D Center, CCCC First Highway Consultants Co., Ltd., Xi’an 710065, China; liujp09@gmail.com; 2 Department of Civil Engineering, University of Louisiana at Lafayette, Lafayette, LA 70504, USA; xsun@louisiana.edu (X.S.); mxs1278@louisiana.edu (M.S.)

**Keywords:** bi-level programming model, full observability, route flow, greedy algorithm

## Abstract

Utilizing the data obtained from both scanning and counting sensors is critical for efficiently managing traffic flow on roadways. Past studies mainly focused on the optimal layout of one type of sensor, and how to optimize the arrangement of more than one type of sensor has not been fully researched. This paper develops a methodology that optimizes the deployment of different types of sensors to solve the well-recognized network sensors location problem (NSLP). To answer the questions of how many, where and what types of sensors should be deployed on each particular link of the network, a novel bi-level programming model for full route observability is presented to strategically locate scanning and counting sensors in a network. The methodology works in two steps. First, a mathematical program is formulated to determine the minimum number of scanning sensors. To solve this program, a new ‘differentiating matrix’ is introduced and the corresponding greedy algorithm of ‘differentiating first’ is put forward. In the second step, a scanning map and an incidence matrix are incorporated into the program, which extends the theoretical model for multiple sensors’ deployment and provides the replacement method to reduce total cost of sensors without loss of observability. The algorithm developed at the second step involved in two coefficient matrixes from scanning map and incidence parameter enumerate all possibilities of replacement schemes so that cost of different combination schemes can be compared. Finally, the proposed approach is demonstrated by comparison of Nguyen-Dupuis network and real network, which indicates the proposed method is capable to evaluate the trade-off between cost and all routes observability.

## 1. Introduction

The network sensor location problem (NSLP) to determine traffic volumes and monitor traffic network status has been of ever-growing interest as the variety of sensor technologies has increased and matured. Different types of sensors can provide different traffic data ranging from point-data to continuous path-data. Nowadays, there are mainly two functional categories of traffic sensors: counting sensors (inductive loop detectors, magnetic sensors, etc.) and scanning sensors (video detection systems, automatic vehicle identification sensors, etc.). Counting sensors, especially inductive loop detectors, have been extensively installed in roadways, where they are able to provide point-data including vehicle counts, presence, occupancy and speed. Scanning sensors can extract the path-data of travel time, route flow and O-D flow in addition to point-data. As for the network, traffic sensors are used to observe and identify critical flow information with regard to route flow, link flow and O-D flow. 

**Figure 1:**
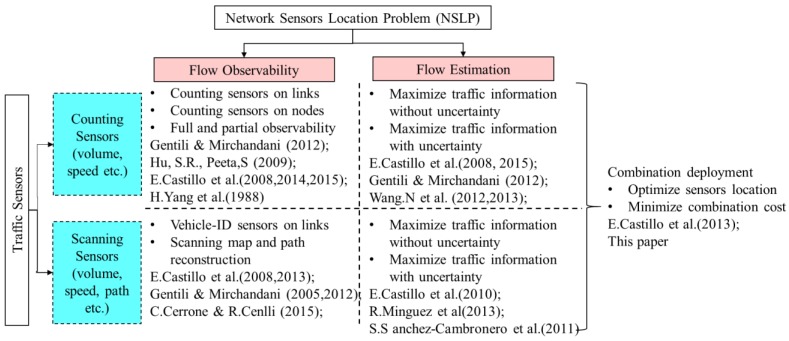
Classification of sensor location problem in the traffic network.

Gentili and Mirchandani [1] reviewed the NSLP in details and categorized this problem into two main classes: (i) Sensor Location Flow-Observability Problem: locating sensors to fully and uniquely observe flow volumes on the network through linear independent equations associated with network topology incidence matrix; (ii) Sensor Location Flow-Estimation Problem: locating sensors to estimate flow volumes on the network based on prior information. Castillo et al. [2] summarized the state-of-the-art literature review and distinguished further this topic into flow observability, estimation and prediction problems based on the different constraints, objective functions and variables. Figure 1 provides an overview of traffic sensors and NSLP. 

The network sensors location problem tries to answer the questions of how many sensors, what types and where corresponding sensors should be located to observe traffic flow to get as much traffic information as possible. There exist different definitions related to observability in NSLP and traffic state estimation. The flow observability in NSLP denotes whether the unknown or unobserved flows can be uniquely calculated in terms of the known or observed flows in a network based on the traffic conservation equations [2]. In general, the flows include route flows, links flows and O-D flows. In practice, the known or observed flows are identified by different traffic detectors. The number and location of sensors could generate the full and partial observable problem depending on if there exists a unique solution of the linear equation system between unknown flow and deployed links. If the equations have a unique solution, we say the network system is fully observable, otherwise it is partially observable (see Section 2). In addition, the observability problem varies from counting sensors to scanning sensors because of different extracted traffic information. As shown in Figure 1, most of the literature concentrated on the single type of sensors (counting or scanning) location problem. Castillo et al [3] proved it is impossible to identify the full route flow under some particular conditions, even if every link is deployed by counting sensors, because link equations are dependent. Castillo et al. [4,5,6,7,8] provided a series of theoretical models and methodology for the flow observability problem related to both types of sensors, including matrix tools, topology methods, objective functions, constraints and algorithm design. Cerrone et al. [9] modified the model of vehicle-ID sensor location for route flow recognition by adding the time order constraint and providing greedy and tabu search algorithm. Mínguez et al. [10] developed the observability and estimation formulation of vehicle-ID sensor location and numerical experiments were performed. Xu et al. [11] developed the optimal model of NSLP for complete link flow observability under uncertainty through considering the error propagation and accumulation. 

Link flow, O-D flow and route flow are dependent (see Section 2). If all of the route flow can be observed, the O-D and link flow can be derived from the linear equation system. Compared with the counting sensors that cannot provide location information of different time, the capacity of scanning sensors to record time stamp and license plate has attracted wide attention on routes flow identification. Transportation agencies hope as much critical traffic information as possible can be obtained from sensors. However, it’s impossible to deploy a mass of scanning sensors in the network, especially for a large network, because of limited budget and resources. Moreover, scanning sensors have a much higher price (at least three times) than counting sensors, for example, the price of an inductive loop varies from $600 to $900 and the price of a video detection system varies from $2400 to $6000 [12]. This noticeable cost effect encouraged us to explore a new method to balance cost and traffic information. The approach of this paper combines counting and scanning sensors to obtain full route flows. To the best of the authors’ knowledge, only Castillo et al. [3] and Fu et al. [13] have paid attention to the combination deployment problem, but the former focused on one feasible solution and the latter aimed at path reconstruction. 

In this paper, we develop a novel bi-level programming model to implement the combination deployment of different types of sensors to observe all routes flow. The first-level obtain the optimized location scheme of scanning sensors based on programming model of multiple constraints by running the “differentiating-first” greedy algorithm. In the second level, we use scanning map and incidence matrix tool to enumerate all scenarios that scanning sensors were replaced by countering sensors under the premise of full routes flow. Finally, the model and algorithm were applied to a Nguyen-Dupuis network and a real network by MATLAB. The results indicate that the proposed approaches can effectively balance between total cost constraints and all routes flow information.

The remainder of this paper is organized as follows: Section 2 presents the counting and scanning sensors observability problem. Section 3 provides the bi-level theoretical model for combination deployment. Section 4 analyzes the algorithm of a different level. Section 5 demonstrates the feasibility and availability of proposed approaches. In the end, conclusion and remarks were summarized in Section 6.

## 2. Sensors Location Observability Problem 

In this section, the flow observability problem in a network is presented with emphasis on comparing the characteristics of single type of sensors and introducing the different equation systems between scanning and counting sensors.

### 2.1. Conservation Laws of Traffic Flows 

Traffic networks consist of nodes and links whose topology can be defined as N = (V, A), where the set of nodes V represents the intersections and the set of links A represents roads through joining node pairs. The users generate different O-D pairs from a given set of origins to a given set of destination and different routes R through traveling a set of roads. Route flow, link flow and O-D flow on a network lead to the following equation system:

(1) $$v_{a}=\sum_{k\in R}\varphi_{ak}r_{k};\;\forall a\in A$$

(2) $$t_{i}=\sum_{k\in R}\eta_{ik}r_{k};\;\forall i\in OD$$

where $\varphi_{ak}$ is the link-route incidence matrix and $\eta_{ik}$ is the route-*OD* incidence matrix. $\varphi_{ak}$ is equal to 1 if route *k* passes through link *a*, and 0 otherwise. $\eta_{ik}$ is equal to 1 if route *k* passes through link *a*, and 0 otherwise. Equation (1) describes the link flow $v_{a}$ is equal to the sum of all routes flow passing through link *a*. Equation (2) states the O-D flow $t_{i}$ is the sum of routes flow passing through this O-D pair.

When the equation system can uniquely determine all unknown route flows, we say this system is fully observable. When the equation coefficient matrix is not full-rank and only some of unknown route flows can be determined by the equation system, we say this system is partially observable; in this case, the location of sensors generate the flow estimation problem based on the prior information [1]. Note the definition of observability is different from the traffic state observability [14]. The partial observability and flow estimation problem could derive from the limitation of the budget (there are not enough sensors for a given network) and dependent linear equations [15,16,17,18]. 

We note that, theoretically, all the flow information (link flow, route flow and O-D flow) of a given network can be obtained from the linear combination of Equations (1) and (2) if all route flows are uniquely determined by sensors. Therefore, we can focus on considering how to locate sensors to monitor each routes’ flow. 

### 2.2. Counting Sensors Observability

In the following part of Section 2, sensors observability problem is presented through comparing the different characteristics between counting sensors and scanning sensors. For simplifying our illustration, we consider a model example with five nodes and seven links.

**Figure 2:**
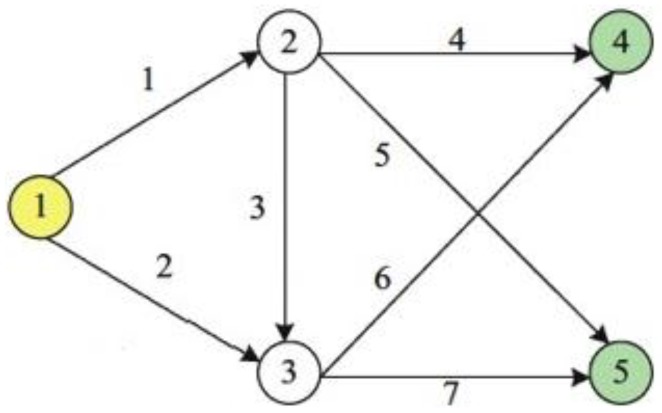
A toy example with 5 nodes and 7 links.

Counting sensors predominate in traffic information detection. This type of sensors can extract the point-information such as speed, occupancy and volume. When a counting sensor is located on the link a, we assume it can identify vehicle volume (ν_a_) of link a in the fixed time interval. As shown in Figure 2, there are 2 O-D pairs, namely t_1_ = (1, 4) and (1, 5). The first O-D pair can be connected by *r*_1_ = {1, 4}, *r*_2_ = {1, 3, 6}and *r*_3_ = {2, 6}, and *r*_2_ = {1, 4}, *r*_4_ = {1, 3, 6}and *r*_5_ = {2, 6} connect the second O-D pair. Table 1 shows all the enumeration information with regard to O-D pairs, routes and links. 

Assume we are interested in monitoring route flow. To this end, we need to answer the following question: how many and where counting sensors should be located on the links? For illustrating this observability problem, we assume to locate three counting sensors on the links 1, 3 and 4, then the following equation can be derived from the link-route connecting relationship:

(3) $$\begin{array}{c} \left(link\;1\right):\;r_{1}+r_{2}+r_{4}+r_{5}=v_{1}\\ \left(link\;3\right):\;r_{2}+r_{5}=v_{3}\\ \left(link\;4\right):\;r_{1}=v_{4} \end{array}$$

By solving the above equation system, $r_{1}$ and $r_{4}$ can be uniquely determined, namely $r_{1}=v_{4}$ and $r_{4}=v_{1}-v_{3}-v_{4}$. But $r_{2}$ and $r_{5}$ cannot be determined because they are dependent in this equation system. If prior information can be obtained from experience and long-term statistics on a network, for example $r_{2}^{0}$ and $r_{5}^{0}$ , we can estimate the optimal values of $r_{2}\;,r_{5}$ through $r_{2}+r_{5}=v_{3},\;\left|r_{2}-r_{2}^{0}\right|$ and | $r_{5}$–$r_{5}^{0}|$.

Consider the extreme scenario that all the links are located by counting sensors, the corresponding equation system is shown as follows:

(4) $$\begin{array}{c} link\;1:\\ link\;2:\\ link\;3:\\ link\;4:\\ link\;5:\\ link\;6:\\ link\;7: \end{array}\left(\begin{array}{cccccc} 1 & 1 & 0 & 1 & 1 & 0\\ 0 & 0 & 1 & 0 & 0 & 1\\ 0 & 1 & 0 & 0 & 1 & 0\\ 1 & 0 & 0 & 0 & 0 & 0\\ 0 & 0 & 0 & 1 & 0 & 0\\ 0 & 1 & 1 & 0 & 0 & 0\\ 0 & 0 & 0 & 0 & 1 & 1 \end{array}\right)\left(\begin{array}{c} r_{1}\\ r_{2}\\ r_{3}\\ r_{4}\\ r_{5}\\ r_{6} \end{array}\right)=\left(\begin{array}{c} v_{1}\\ v_{2}\\ v_{3}\\ v_{4}\\ v_{5}\\ v_{6}\\ v_{7} \end{array}\right)$$

Echelon form:

(5) $$\left(\begin{array}{cccccc} 1 & 0 & 0 & 0 & 0 & 0\\ 0 & 1 & 0 & 0 & 1 & 0\\ 0 & 0 & 1 & 0 & 0 & 1\\ 0 & 0 & 0 & 1 & 0 & 0\\ 0 & 0 & 0 & 0 & 1 & 1\\ 0 & 0 & 0 & 0 & 1 & 1\\ 0 & 0 & 0 & 1 & 0 & 0 \end{array}\right)\left(\begin{array}{c} r_{1}\\ r_{2}\\ r_{3}\\ r_{4}\\ r_{5}\\ r_{6} \end{array}\right)=\left(\begin{array}{c} v_{4}\\ v_{3}\\ v_{2}\\ v_{5}\\ v_{7}\\ v_{3}-v_{6}+v_{2}\\ v_{1}-v_{3}-v_{4} \end{array}\right)$$

The 7 × 6 coefficient matrix in Equation (4) is the link-route incidence matrix. We can obtain the R solution by Gaussian elimination method and write it as the reduced row echelon form (as shown in Equation (5)). The equation system contains seven known variables and six unknown variables, but the rank of a coefficient matrix is equal to 5 from Equation (5), namely it is a non-full rank matrix. In other words, there is one unknown variable ($r_{6}$) that cannot be uniquely determined by the equation system. The reason is there exists a dependent relationship between the known variables. The dependent relationship (bold rows: $v_{5}=v_{1}-v_{3}-v_{4}\;\mathrm{and}\;v_{7}=v_{3}-v_{6}+v_{2}$) are discovered from Equation (5). The above example has indicated that full routes flow cannot be identified by counting sensors in some special situations, even if all the links are located by this type of sensors.

### 2.3. Scanning Sensors Observability

The most notable feature of scanning sensors is recording the time stamp and license plate of a vehicle passing it, which can provide continuous link information. A single vehicle can be identified at a different time on a given network. Consider the same example in counting sensors. For example, the license plate of a vehicle is scanned at time µ1 on link 2, as shown in Table 1, we can say this vehicle is traveling $r_{3}$ or $r_{6}$ to realize the trip from origin node 1 to destination node 4 or 5. when this vehicle is scanned at time µ2 (µ2 = µ1 + ∆ > µ1) on link 6, we can completely determine the vehicle is using $r_{3}$ from origin node 1 to destination node 4.

Considering the links (2, 3, 6) to locate scanning sensors in the Figure 2 network, the corresponding linear equation system at a different time is as follows:

(6) $$\begin{array}{c} link\;\left(2\right):\;r_{3}+r_{6}=v_{2}\\ link\;\left(3\right):\;r_{2}+r_{5}=v_{3}\\ link\;\left(6\right):\;r_{2}+r_{3}=v_{6}\\ link\;\left(2,\;6\right):\;r_{3}=v_{2,\;6}\\ link\;\left(3,\;6\right):\;r_{2}=v_{3,\;6} \end{array}$$

The first three equations are determined by a single sensor, in fact, there is no difference between a counting sensor and a scanning sensor in this situation. If the same links are deployed by counting sensors, only the first three equations can be obtained. However, the last two equations are joint recognition equation through scanning two links due to the fact we are recording license plate and time stamp. By solving this equation system, $r_{2}$, $r_{3}$, $r_{5}$ and $r_{6}$ flows can be uniquely determined, which is impossible for counting sensors to obtain four unknown variables through three linear equations.

Again, consider the same links (1, 3, 4) in counting sensors example to deploy scanning sensors. The corresponding equation system can be obtained from the Table 1:

(7) $$\begin{array}{c} link\;\left(1\right):\;r_{1}+r_{2}+r_{4}+r_{5}=v_{1}\\ link\;\left(3\right):\;r_{2}+r_{5}=v_{3}\\ link\;\left(4\right):\;r_{1}=v_{4}\\ link\;\left(1,\;3\right):\;r_{2}+r_{5}=v_{1,\;3}\\ link\;\left(1,\;4\right):\;r_{1}=v_{1,\;4} \end{array}$$

By solving Equation (7), the solution is the same as that of Equation (3), namely deploying counting sensors on the links (1, 3, 4). The result indicates that, in some situations, the scanning sensors can be replaced by counting sensors without sacrificing information. In addition, we can conclude there exist the optimal scheme for locating sensors on a given network under the precise of same sensors number through comparing Equations (6) and (7). 

Comparing the above examples, the following differences between counting sensors and scanning sensors are noticeable: (i) scanning sensors provide more information than counting sensors for the same number and location, which can be translated into linearly independent equations; (ii) when adding (removing) a link to be monitored by sensors, counting sensors only add (remove) one linear equation to/from the old equation system and a scanning sensor adds (removes) at least one linear equation (involved with all routes in the link) into an old equation system; (iii) scanning sensors provide the joint recognition equation from multiple scanned links, that is scanning sensors not only ***cover*** route flows, but also ***differentiate*** route flows. At this point, the following problem arises: what is the minimum cost of sensors when combining counting and scanning sensors and where to locate them respectively, such that a traffic network system is fully observable?

## 3. Bi-Level Mathematical Model

In this section, a bi-level mathematical model is proposed to answer the problem described in Section 2. The first level uses the scanning map technique [5] to determine the near-optimal locations of scanning sensors on a given network. The second level uses the matrix tool to answer how to replace scanning sensors into counting sensors so that the total cost of the combination is minimum.

### 3.1. The First Level Model

The level focuses on how to deploy scanning sensors so that all routes flow can be uniquely determined. For that, we first introduce the scanning map definition [5,18,19].

Let N=(V,A) be a network and R be a set of routes on the network. Given a location of scanning sensors on a subset U⊆A of links, we can define a scanning map $SM\left(U\right)=\left\{C_{R_{i}}:\;C_{R_{i}}=U\cap R_{i},\;i=1,\;2,\;3,\ldots ,\left|R\right|\right\}$, where each scanning set $C_{R_{i}}$ is associated with route $R_{i}$ that its links are scanned by sequential order. If all the scanning sets in SM are not empty and not repeating a subset, namely $C_{R_{i}}\neq \emptyset$ and $C_{R_{i}}\neq C_{R_{j}}$ for each i≠j=1, 2,…, |R|, then scanning map set can uniquely determine all routes flow and the network is a fully observable system [1].

Consider again the example in Figure 2. Assume three scenarios that links (1, 2, 3, 4,), links (1, 2, 4, 6), and links (1, 2, 3, 4, 6) are respectively deployed by scanning sensors, that is $U_{1}=\left\{1,\;2,\;3,\;4\right\}$, $U_{2}=\left\{1,\;2,\;4,\;6\right\}$ and $U_{3}=\left\{1,\;2,\;3,\;4,\;6\right\}$. The responding scanning map set is shown in Table 2.

Table 2 indicates that $U_{1}$ covering all routes can uniquely determine $r_{1}$, $r_{4}$, but it cannot differentiate $r_{2}$, $r_{5}$ and $r_{3}$, $r_{6}$. $r_{1}$, $r_{2}$, $r_{3}$ and $r_{6}$ can be uniquely differentiated by scheme $U_{2}$, which illustrates the location of sensors has significant effect on differentiating routes when covering the same number of routes. Comparing $U_{1}$ and $U_{2}$, there is only one different link (link 3 in $U_{1}$ and link 6 in $U_{2}$), but the number of unique identified route flow varies dramatically. $U_{3}$ can uniquely identify all routes flow in the network when adding one link to be scanned into $U_{1}$ and $U_{2}$. The example implies that scanning sensors provide traffic flow information through covering routes and differentiating routes. In addition, different links have a varying weight to differentiate routes, which provides the direction of selecting observed links. There must exist a feasible scanning map to observe the full route flow. Therefore, the first level mathematical model locating scanning sensors to observe the full routes flow is as follows:

(8) $$min\sum_{a\in A}x_{a}$$

(9) $$s.t.\;\sum_{a\in A}\lambda_{r_{i}}^{a}x_{a}\geq 1\;\forall r_{i}\in R$$

(10) $$\sum_{a\in A}\left(\lambda_{r_{i}}^{a}+\lambda_{r_{j}}^{a}\right)\left(1-\lambda_{r_{i}}^{a}\lambda_{r_{j}}^{a}\right)x_{a}\geq 1\;\forall r_{i}\neq r_{j}\in R$$

(11) $$x_{a}\in \left\{0,\;1\right\}$$

where $x_{a}$ is a binary variable associated with each link a∈A and is equal to 1 when the scanning sensor is deployed on link a, 0 otherwise.$\lambda_{r_{i}}^{a}$ is link-route incidence parameter and is equal to 1 when route $r_{i}$ uses link a, 0 otherwise.

The objective function (8) is minimizing the sum of scanning sensors to be deployed on a network. Constraints (9) cover constraints that ensure each route is scanned by at least one link with a scanning sensor. These constraints guarantee all routes can be covered by scanning sensors. Constraints (10) are differentiating constraints that ensure each route is differentiated by at least one different link with a scanning sensor. $\left(\lambda_{r_{i}}^{a}+\lambda_{r_{j}}^{a}\right)\left(1-\lambda_{r_{i}}^{a}\lambda_{r_{j}}^{a}\right)$ ensure that it is equal to 1 only if link a is in only one of route $r_{i}$ or $r_{j}$, 0 otherwise. These constraints guarantee all routes can be differentiated by scanning sensors. Combining with the scanning map concept, constraints (9) ensure that all the scanning sets in SM are not empty ($C_{r_{i}}\neq \emptyset$). Constraints (10) ensure that all the scanning sets in SM are not repeating a subset ($C_{r_{i}}\neq C_{r_{j}}$). Therefore, we can obtain the feasible near-optimal solutions by solving the programming model so that all routes flow can be uniquely determined, and then all flow information can be derived from Equations (1) and (2).

### 3.2. The Second Level Model

This level model aims at answering how to replace scanning sensors into counting sensors without loss of flow information, namely how to implement the combined deployment of two types of sensors in a network so that the total cost is minimum under the premise of full route flow observability. Assume each link has only one sensor. For illustration, consider again the example in Figure 2 and assume $U_{1}=\left\{1,\;2,\;3,\;4,\;6\right\}$, $U_{2}=\left\{2,\;3,\;6\right\},\;U_{3}=\left\{3,\;6\right\},\;U_{4}=\left\{1,\;3,\;4\right\}$, $U_{5}=\left\{4\right\}$ and the responding scanning map set is shown in Table 3.

As shown in Table 3, $U_{1}$ is covering and differentiating all routes. When scanning sensors on links 2 and 6 are removed from $U_{1}$, *r*_1_, *r*_2_, *r*_4_ and *r*_5_ can be covered by $U_{4}$, but *r*_2_ and *r*_5_ cannot be differentiated because of existing repeat subset in $SM\left(U_{4}\right)$. Even if counting sensors are added on other links, we think this situation cannot identify all routes because it cannot record time stamp and license plate (introduced in Section 2). When scanning sensors in link 1 and 4 are removed from $U_{1}$, $U_{2}$ can cover and differentiate *r*_2_, *r*_3_, *r*_5_, *r*_6_. Similarly, the part of routes flow can be covered and uniquely differentiated by $U_{3}$ and $U_{5}$ ($U_{3}\to \{r_{2},r_{3},r_{5}\},\;U_{5}\to \{r_{1}\}$). The following problems arise: whether the rest of routes are uniquely determined by counting sensors? If there exist feasible replacement schemes, what requirements are satisfied?

For answering the above questions, the corresponding equations that known routes by scanning map and links with scanning sensors are excluded from equation system (4) are as shown in Table 4. The gray section elements are known by scanning sensors and bold section elements are equations coefficients between unknown routes and links. The route flow observability problem for rest parts (bold section) can be translated into counting sensors full observability in Section 2.2. As introduced in Section 2, when link-route incidence matrix is full rank, the corresponding routes flow can be uniquely determined by counting sensors. The unknown variables are $r_{1}$, $r_{4}$ in $U_{2}$ scheme. The matrix rank of corresponding equations for rest parts is equal to 2, therefore full route flow can also be uniquely determined by replacing scanning sensors on link 1, 4 in $U_{1}$ with counting sensors on link (1, 4 or 1, 5 or 4, 5). In the same way, the corresponding rank of $U_{3}$ is equal to 3, and equal to the number of unknown routes, then we can say this replacement solution is feasible for full routes flow. However, when scanning sensors on link 1, 2, 3 and 6 are removed from $U_{1}$, the corresponding rank of $U_{5}$ is equal to 3, less than the number of unknown routes (5), so this replacement solution is not feasible for full route flows. 

Based on the above truth, we can define the requirements of full route flows through a matrix tool. When two types of sensors exist on a network at the same time, $V_{s}$ is a set of links with scanning sensors and $V_{sw}$ is a set of scanning in $SM\left(V_{s}\right)$. $V_{c}$ is a set of links with counting sensors. $V_{s}$ and $V_{c}$ are not repeating a subset of links, namely $V_{s}\cap V_{c}=\emptyset$. $H_{s}$ is the equation coefficient matrix from $SM\left(V_{s}\right)$ and $H_{c}$ is the equation coefficient matrix from $V_{c}$-route parameter. $R_{s}$ and $R_{c}$ are the set of routes by $V_{s}$ and $V_{c}$ respectively, and $R_{s}\cup R_{c}=R$ and $R_{s}\cap^{\text{​}}R_{c}=\emptyset$. Therefore, the routes formulation can be expressed as follows:

(12) $$\left(\begin{array}{c} H_{s}\;0\\ -\;-\\ 0\;H_{c} \end{array}\right)\left(\begin{array}{c} R_{s}\\ R_{c} \end{array}\right)=\left(\begin{array}{c} V_{sw}\\ -\\ V_{c} \end{array}\right)$$

When the rank of $H_{s}$ and $H_{c}$ are full rank at the same time, we can say all route flows in a network can be uniquely determined by the combined deployment of scanning and counting sensors. Therefore, the second level mathematical model can be stated as follows:

(13) $$min\sum_{a\in A}c_{1}x_{a}+\sum_{b\neq a\in A}c_{2}y_{b}$$

(14) $$s.t.\;\sum_{a\in A}\lambda_{r_{i}}^{a}x_{a}+\sum_{b\neq a\in A}\lambda_{r_{k}}^{b}y_{b}\geq 1\;\forall r_{i}\neq r_{k}\in R$$

(15) $$\sum_{a\in A}\left(\lambda_{r_{i}}^{a}+\lambda_{r_{j}}^{a}\right)\left(1-\lambda_{r_{i}}^{a}\lambda_{r_{j}}^{a}\right)x_{a}+\xi_{c}\sum_{b\neq a\in A}\lambda_{r_{k}}^{b}y_{b}\geq 2\;\forall r_{i}\neq r_{j}\in R_{s},\;\forall r_{k}\neq r_{i}\in R_{c}$$

(16) $$x_{a},y_{b}\in \left\{0,\;1\right\}$$

(17) $$R_{s}\cup R_{c}=R\;R_{s}\cap R_{c}=\emptyset$$

where $x_{a}$, $\lambda_{r_{i}}^{a}$ are the same definition as the first level. $y_{b}$ is a binary variable associated with link b∈A and is equal to 1 when counting sensor is deployed on link b, 0 otherwise. $c_{1}$ and $c_{2}$ are the cost constant of scanning and counting sensors respectively. $\lambda_{r_{k}}^{b}$ is link-route parameter of counting sensors and is equal to 1 when route $r_{k}$ uses link b, 0 otherwise. $\xi_{c}$ is binary variable regarding matrix $H_{c}$ in Equation (12) and is equal to 1 when $H_{c}$ is a full rank matrix, 0 otherwise. 

The objective function (13) minimizes the combined cost of scanning and counting sensors. Constraints (14) are joint coverage constraints that ensure all routes can be covered by sensors, irrespective of types, namely $R_{s}\cup R_{c}=R$ in Equation (12). The left half of joint differentiating constraints (15) ensures that links with scanning sensor can differentiate $R_{s}$ (i.e., $H_{s}$ is a full rank matrix). $\xi_{c}\sum_{b\neq a\in A}\lambda_{r_{k}}^{b}y_{b}$ in Equation (15) guarantees that the remainder of the routes can be uniquely determined by links with counting sensors (i.e., $H_{c}$ is full rank matrix). 

## 4. Algorithm for Each Level Model

It has been proved that these types of models in first level are computationally NP-hard [20,21,22]. Therefore, in this Section, a greedy algorithm of differentiating first is put forward to obtain near-optimal solution in the first level model. Then a heuristic algorithm is presented to solve the second level model. Finally, we can obtain the feasible and near-optimal approaches for combination deployment. 

### 4.1. Greedy Algorithm for First Level Model 

We have defined two types of constraints as covering and differentiating. The above-mentioned example denotes the differentiating constraints play a more important role in identifying routes number. Based on this truth, the greedy algorithm considering ‘differentiating first’ principle is proposed to solve the first level model. This approach is inspired by research [9]. In this algorithm, there are two criteria:

Differentiating criterion: Differ(U,a) is the sum of additional routes that can be uniquely differentiated by link a when adding scanning sensors on link a (U is a set of links with scanning sensors). 

Covering criterion: Cover(U,a) is the sum of additional routes that can be covered by link a when adding scanning sensors on link a.

**Figure 3:**
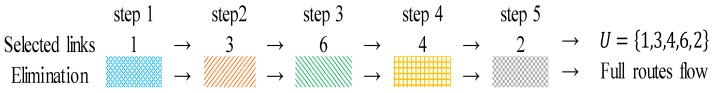
The procedure of selecting links and eliminating rows in greedy algorithm.

The ‘differentiating first’ principle is prioritizing to select the link to be scanned that can differentiate the maximum routes through ranking Differ(U,a). Consider the example again in Figure 1. Table 5 shows the process of a greedy algorithm considering ‘differentiating first’ principle. The absolute values of difference between two random rows in link-route incidence matrix stand for the differentiated routes by different links. The absolute value is equal to 1 when link can differentiate specific routes, 0 otherwise. For example, $v_{5}$ can differentiate $r_{1}$ and $r_{4}$, $r_{2}$ and $r_{4}$, $r_{3}$ and $r_{4}$, $r_{4}$ and $r_{5}$, $r_{4}$ and $r_{6}$ through the subtraction of two random rows. Similarly, the differentiate features of other links can be calculated and the ***initial differentiating matrix*** can be presented (Table 5). First step, let $U=U_{0}=\emptyset$ and select the link 1 with maximum summation (8) of column in initial differentiating matrix to deploy scanning sensors, then eliminate all rows (pattern of step 1 in Figure 3) of $v_{1}=1$ in initial differentiating matrix. At this moment, $U=U_{1}=\left\{1\right\}$ and changeable differentiating matrix consists of the rest rows of initial differentiating matrix, and select the link 3 with maximum (4) of differentiating routes to deploy scanning sensors. Repeat the above steps till differentiating matrix is empty and $U=U_{4}=\left\{1,\;3,\;4,\;6\right\}$. So far, the differentiating routes process is finished, and the next step is checking whether all routes are covered by selecting links or not. In this example, $r_{6}$ is not covered by $U_{4}=\left\{1,\;3,\;4,\;6\right\}$ and the rest links are 2, 5, 7. In the same way, select the link 2 with maximum summation (1) of column in changeable differentiating matrix. Finally, one feasible solution $U=U_{5}=\left\{1,\;3,\;4,\;6,\;2\right\}$ is obtained so that all routes can be uniquely determined. 

Based on the above process, the greedy algorithm for solving first level model can be expressed as follows:

INPUT: link-route incidence matrix of traffic network topology.
OUTPUT: set of links to be scanned by scanning sensors.
Step 1: Initialization-U is the set of selected links and H is link-route incidence matrix.
U=∅;
Step 2: Initial differentiating matrix (H_differ)
generate H_differ by subtraction of two random rows in H.
Step 3: Greedy process
While H_differ is not empty
select the link a∈A\U with maximum of Differ(U,a);
eliminate all rows involved with link a;
Step 4: Check coverage
While H is not empty
select the link a∈A\U with maximum of Cover(U,a);
Step 5: Return U

### 4.2. Algorithm for Second Level Model 

Equation (12) provides the key point of algorithm for the second level model. Let $U=U_{s}\cup U_{c}$, $U_{s}\cap U_{c}=\emptyset$. $U_{s}$ is the set of links with scanning sensors and $U_{c}$ is the set of links with counting sensors. N_re is the number of scanning sensors replaced by counting sensors. Consider the example in Figure 1 again. The process of replacement is shown in Table 6.

As shown in Table 6, all possibilities of the replacement scheme are enumerated. When the requirement of full rank is satisfied, $H_{s}$ and $H_{c}$ are equal to 1, 0 otherwise. It’s redundant to judge if $H_{c}$ is true or not, when $H_{s}$ is false. The bold parts are feasible combination schemes. There exist serval replacement schemes for a given original scanning location and exist different replaced links when the number of scanning sensors are fixed. In this process, the possibilities of combination iteration are $C_{5}^{1}=5,C_{5}^{2}=10,\;C_{5}^{3}=10$ respectively for N_re = 1, 2, 3. When N_re = 4, 5, full rank conditions are false, so the maximum of replaced scanning sensors is 3 in this example. Comparing the cost of different sachems, the maximum cost ($5c_{1})$ of single scanning sensors is reduced gradually with increase of replaced links (when N_re = 1, 2, 3, the combination cost is $4c_{1}+c_{2},3c_{1}+2c_{2}$ and $2c_{1}+3c_{2}$ respectively ($c_{1}>c_{2})$). 

Therefore, based on the feasible solution from the first level model, the possible combination schemes of minimizing combination cost can be searched by the following algorithm:

INPUT: link-route incidence matrix of traffic network topology and set of links with scanning sensors for full routes flow observability.

OUTPUT: combination deployment schemes–set of links by scanning sensors and set of links by counting sensors.

Step 1: Initialization-U is the set of selected links with scanning sensors and H is link-route incidence matrix. n is the number of removed scanning sensors. $U_{left}$ is the rest links in initial *U* after removing n links.

U = the result of first level.

n = 1.

Step 2: Replacement process

While n≤ length of U

select randomly n (n≤ length of U) links to be removed from U and generate $U_{left}$;

perform scanning map by $U_{left}$ and generate corresponding $H_{s}$ and $H_{c}.$

if $H_{s}$ and $H_{c}$ are full-rank, record the corresponding links and location of matrix element, and $U_{s}=U_{left},\;U_{c}$ is set of links that make $H_{c}$ meet the requirement of full rank. This step can be finished by identify matrix.

Step 3: Return $U_{s}$ and $U_{c}$.

## 5. Numerical Experiment

In this section, the proposed approach is applied to two more complicated networks for demonstrating its feasibility and availability. All algorithms are implemented using MATLAB.

### 5.1. Nguyen-Dupuis Network

**Figure 4:**
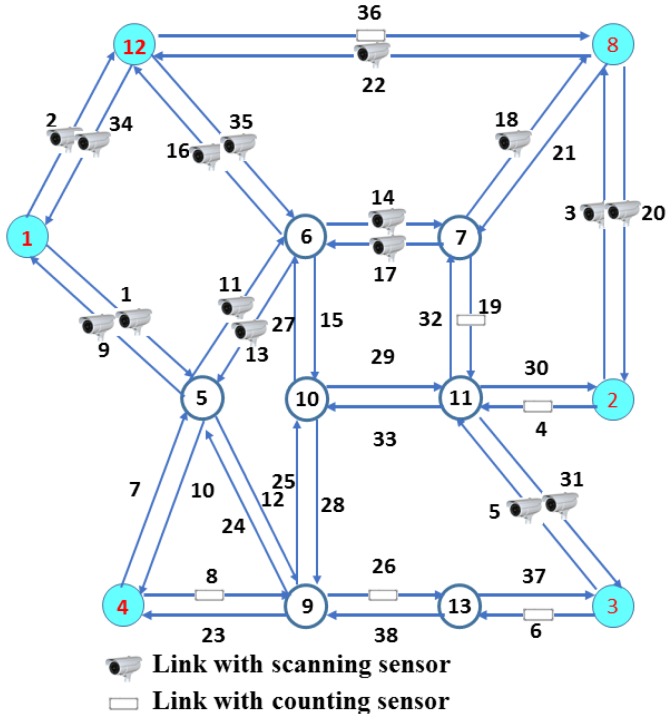
The topology structure of Nguyen-Dupuis network [5] and a feasible combination deployment scheme with 16 scanning sensors and six counting sensors.

The Nguyen-Dupuis network is used as an example in many similar studies and consist of 13 nodes and 38 links [5]. The network topology can be seen in Figure 4 and the routes, O-D pairs and links relationship can be seen in Table 7. Totally, there exist 18 O-D pairs and 50 routes in the Nguyen-Dupuis network. Based on the network topology, the corresponding incidence matrix can be generated, then the corresponding algorithm of bi-level model is implemented respectively. The result of bi-level model for full routes flow observability of Nguyen-Dupuis network can be obtained:

*First level* (single scanning sensors): using the greedy algorithm considering ‘differentiating first’ principle, the set of links to be scanned by scanning sensors is obtained (cpu = 0.061s):

U={14, 17, 29, 33, 22, 36, 6, 26, 16, 35, 3, 20, 5, 31, 2, 34, 1, 9, 4, 11, 13, 18}

This algorithm provides a near optimal and feasible solution of 22 scanned links. The number of scanned links is similar to the previous studies in Nguyen-Dupuis network. However, the location of selected links is different from other researches [3,5], which indicates there exists a variety of layout schemes and different algorithms may generate different feasible solutions.

*Second level* (combining sensors): using the algorithm in Section 4.2, the set $U_{s}$ of links with scanning sensors and set $U_{c}$ of links with counting sensors are generated. The result is shown in Table 8 considering different number of replaced links. 

In this example, the deployment scheme of single scanning sensors is U = {14, 17, 29, 33, 22, 36, 6, 26, 16, 35, 3, 20, 5, 31, 2, 34, 1, 9, 4, 11, 13, 18}. Based on this scheme, the maximum of replaced sensors is equal to six without loss of observability. The different combination schemes of different replaced numbers can be calculated through running the proposed algorithm. The results indicate that there exist various available combination schemes of scanning and counting sensors to observe all route flows. The different combinations refer to the numbers of different types of sensors and replacement locations. Furthermore, different replacement locations could generate more economical schemes, such as the scheme in the first row of left half sheet in Table 8. The six scanning sensors are replaced by four counting sensors, which indicates some locations provide reductant information. It’s critical to optimize the algorithm structure.

### 5.2. Case Study

**Figure 5:**
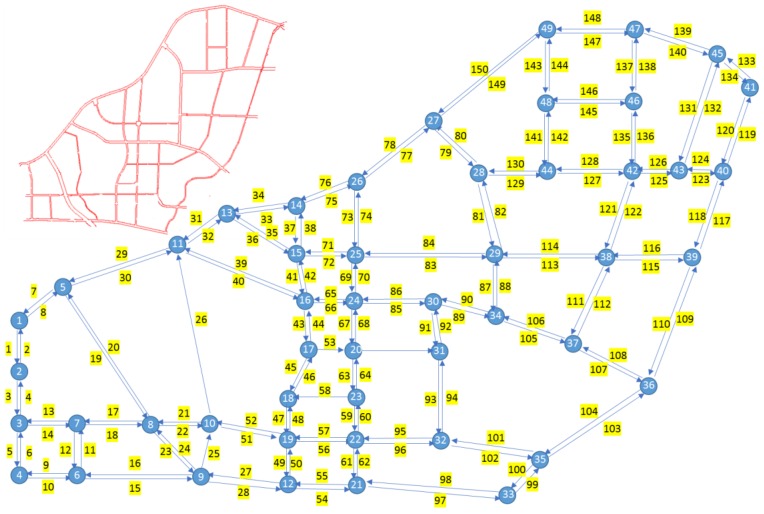
The real network of case study showing topology, links with scanning sensors and links with counting sensors.

In this numerical experiment, a larger size real-world network is selected to demonstrate the proposed approach. The real traffic network (left upper corner in Figure 5) is located at the H-tech district of Xi’an in China and has 49 nodes and 150 links. Except for links 26, 53 and 58 that are one-way links, the others have bi-directional flows between two nodes (as shown in Figure 5). The reason to consider the main routes is that parking is not permitted in many zones and users choose alternative routes as practical as possible rather than randomly. Therefore, in this real network, we consider 176 O-D pairs and 347 routes. It’s a huge budget for a large traffic network to obtain full observability only considering a camera system. In other words, the budget is a critical constraint for extracting sufficient flow information. To illustrate this problem and determine full routes flow, the bi-level programming model as indicated in the previous section is applied to this case study.

The following set of selected links with single scanning sensors is obtained from the first level model and algorithm after cpu 0.461s.

U = {118, 101, 109, 41, 108, 98, 107, 115, 124, 50, 69, 140, 133, 27, 125, 14, 19, 80, 75, 84, 10, 104, 79, 70, 150, 49, 23, 147, 76, 97, 35, 120, 32, 148, 110, 99, 38, 119, 36, 149, 31, 131, 26, 96, 60, 139, 141, 6, 29, 100, 117, 77, 136, 134, 44, 5, 59, 73, 8, 24 ,33, 113, 34, 114, 144, 43, 78, 12, 103 ,137, 11, 4, 13, 39, 20, 81, 123, 132}

When only scanning sensors are considered to extract all routes flow, the near-optimal solution is the set of 79 scanned links, which indicates 53% of the links to be scanned. Compared to the previous in the Cuenca network (the minimum 34% (175) scanned links sufficient for full observability) [3], the result is relatively higher because of the different network topology and route. The truth is important for a much larger network to estimate the cost of full observability. However, accounting for the high cost of scanning sensors and demand of optimizing the budget, the second level model and algorithm are implemented in the real network. 

In this real network, the maximum of replaced scanning sensors is equal to 21 based on the result of second level algorithm. The near optimal combination scheme is shown as follows:

$U_{s}$ = {118, 101, 109, 41, 108, 98, 107, 115, 124, 50, 69, 140, 133, 27, 125, 14, 19, 80, 75, 84, 10, 104, 79, 150, 49, 147, 76, 97, 35, 120, 32, 148, 110, 38, 119, 36, 149, 31, 131, 139, 141, 6, 100, 77 ,136, 134, 59, 8, 24 ,33, 113, 34, 11, 4, 20, 81, 132}

$U_{c}=\{$123, 39, 13, 137, 103, 12, 78, 43, 144, 114, 73, 5, 44, 117, 29, 60, 26, 96, 99, 23, 70}

This scheme consists of 56 scanning sensors and 21 counting sensors. It’s necessary to emphasize this scheme is a relatively optimal feasible solution to observe all routes or routes of interest flow. The feasible schemes are far more than one, especially for a larger traffic network. The computational times increase exponentially with the increase of scanned links in the first level algorithm, therefore this paper only focuses on obtaining one near optimal solution in the real traffic network. 

**Figure 6:**
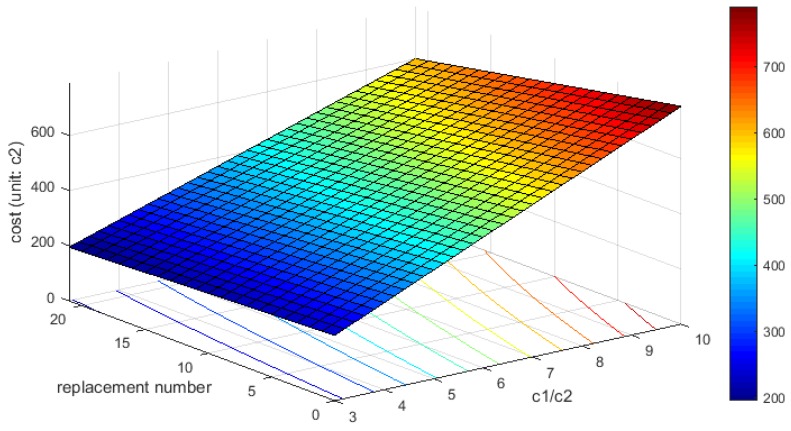
The cost of different combination schemes based on the price of inductive loop.

To illustrate the total cost of different combinations, inductive loop and video detection systems are chosen to represent counting and scanning sensors, respectively. The price of an inductive loop varies from $600 to $900 and the price of a video detection system varies from $2400 to $6000 [12]. Assume $c_{1}$ and $c_{2}$ are the prices of a video detection system and inductive loop, respectively, then $c_{1}/c_{2}$ approximately varies from 3 to 10. $n_{ws}$ and $n_{wc}$ is the number of video detection systems and inductive loops in scheme w, respectively. As shown in Figure 6, the replacement number refers to the different combination schemes and the maximum value is equal to 21. 

The total cost of different combination schemes can be calculated by the following formula:

(18) $$Total\;cost_{ws}=n_{ws}c_{1}+n_{wc}c_{2}=\left(n_{wc}+\frac{c_{1}}{c_{2}}n_{ws}\right)c_{2}$$

As shown in Figure 6, the total cost is monotonously increasing in the horizontal and longitudinal direction. In other words, the total cost decreases as the number of replaced sensors increases and rises with the growth of ratio of $c_{1}$ to $c_{2}$. The most economical combination scheme is composed of 56 scanning sensors and 21 counting sensors, and its total cost is equal to 195 units ($c_{2}$). The deployment scheme of 79 scanning sensors, namely a single type of sensors, is the most expensive and the total cost reaches up to 790 unit ($c_{2}$). The maximum cost is approximately four times higher than the minimum, as shown in the cost distribution diagram. In other words, the total cost of sensors layout for full flow observability is reduced by 75% through implementing the proposed approach in this case study. There exists different reductions because of different network topologies and scales. Obviously, the combination deployment scheme outperforms the scheme using a single type of sensors from a viewpoint of sensor deployment cost. Unfortunately, few previous studies paid attention to combine the multi-type of sensors for observability in their case studies. Therefore, it’s not trivial to optimize and combine different types of sensors to implement full route observability, especially for a larger network. 

It’s necessary to indicate that the proposed approach requires the information of all or interesting routes. It’s critical for the output result that extracting routes information be as practical as possible before generating an input matrix. Again, one feasible near-optimal solution can be obtained from the proposed approach because it is computationally NP-hard. The proposed approach provides many different combination schemes that consist of different types and number of sensors. Considering the maintenance and stability of sensors, the transportation agency can replace old counting sensors with scanning sensors stepwise according to budget constraints, which give lower maintenance costs and more information. 

## 6. Conclusion and Future Research

In this paper, combined deployment of different types of sensors in the network sensors location problem (NSLP) field is addressed. A novel bi-level programming model for full route flows is presented to resolve the problem of combined deployment of scanning and counting sensors. The contributions of this paper are theoretical and algorithmic, that is a programming model in the second level and differentiating matrix in the greedy algorithm are first put forward. Scanning maps and incidence matrices are incorporated into the programming model at the same time, which is also feasible when the first level model doesn’t exist. The reason to present first level model is to provide a feasible input matrix for the second level and compare the total cost between single scanning sensors and combination schemes. 

Using some illustrative examples, this study reveals the essential difference between scanning and counting sensors is in the aspect of extracting network information. Scanning sensors can provide more independent linear equations than counting sensors. It’s impossible for counting sensors in some situations to observe the full flow information even this type of sensors are deployed at all links. In addition, the matrix tool plays a pivotal role in the algorithm process, for example differentiating matrix, covering matrix, the empty matrix in elimination process and full-rank. Traffic sensors provide the flow information through covering and differentiating constraints from the viewpoint of mathematic inequalities. Therefore, covering and differentiating principles are defined in the algorithm process. Examples of different scale illustrate how the algorithm resolves the bi-level programming model, which provides a clear logic method to implement different types of sensors on a given network. 

The proposed approach provides the location set of single scanning sensors and the corresponding location set combinations of scanning and counting sensors. A computational experiment in a real network is performed. The results emphasize the importance of the combined deployment problem, no matter the size of the traffic network. The proposed approach evidently outperforms the layout schemes using a single type of sensor from a viewpoint of total cost, especially for a large network, where the maximum cost is approximately four times higher than the minimum in the case study. 

The study provides directions for implementing different types of sensors to extract full route flows. Transportation agencies can combine and replace sensors in a stepwise way at feasible locations according to management demand and budget through the proposed approaches. However, there are several extensions for future work: (i) it’s critical for a large network to improve the efficiency of the algorithm; (ii) the methods described in this paper focus on the flow information. Future research must be extended to the travel time and reliability of the network; (iii) more work is needed to study dynamic traffic networks because of the changes of traffic information in short time intervals. 

## Figures and Tables

**Table 1:** O-D trips, routes and route links in the Figure 2 example.

OD Pair	Routes (ri)	Links
1, 4	*r*_1_	1, 4
	*r*_2_	1, 3, 6
	*r*_3_	2, 6
1, 5	*r*_4_	1, 5
	*r*_5_	1, 3, 7
	*r*_6_	2, 7

**Table 2:** Scanning map illustration with $U_{1}=\left\{1,\;2,\;3,\;4\right\}$, $U_{2}=\left\{1,\;2,\;4,\;6\right\}$ and $U_{3}=\left\{1,\;2,\;3,\;4,\;6\right\}$.

OD Pair	Routes (*r*_i_)	Links	$SM\left(U_{1}\right)=U_{1}\cap R_{i}$	$SM\left(U_{2}\right)=U_{2}\cap R_{i}$	$SM\left(U_{3}\right)=U_{3}\cap R_{i}$
1, 4	*r*_1_	1, 4	{1, 4}	{1, 4}	{1, 4}
	*r*_2_	1, 3, 6	{1, 3}	{1, 6}	{1, 3, 6}
	*r*_3_	2, 6	{2}	{2, 6}	{2, 6}
1, 5	r_4_	1, 5	{1}	{1}	{1}
	*r*_5_	1, 3, 7	{1, 3}	{1}	{1, 3}
	*r*_6_	2, 7	{2}	{2}	{2}

**Table 3:** Illustration of the process to replace scanning sensor.

OD Pair	Routes (*r*_i_)	Links	$U_{1}\cap R_{i}$	$U_{2}\cap R_{i}$	$U_{3}\cap R_{i}$	$U_{4}\cap R_{i}$	$U_{5}\cap R_{i}$
1, 4	*r*_1_	1, 4	{1, 4}	{-}	{-}	{1, 4}	{4}
	*r*_2_	1, 3, 6	{1, 3, 6}	{3, 6}	{3, 6}	{1, 3}	{-}
	*r*_3_	2, 6	{2, 6}	{2, 6}	{6}	{-}	{-}
1, 5	*r*_4_	1, 5	{1}	{-}	{-}	{1}	{-}
	*r*_5_	1, 3, 7	{1, 3}	{3}	{3}	{1, 3}	{-}
	*r*_6_	2, 7	{2}	{2}	{-}	{-}	{-}

**Table 4:** Corresponding equation coefficients after excluding known variables from Equation (4).

$U_{2}=\left\{2,\;3,\;6\right\}$							$U_{3}=\left\{3,6\right\}$							$U_{5}=\left\{4\right\}$						
	$r_{1}$	$r_{2}$	$r_{3}$	$r_{4}$	$r_{5}$	$r_{6}$		$r_{1}$	$r_{2}$	$r_{3}$	$r_{4}$	$r_{5}$	$r_{6}$		$r_{1}$	$r_{2}$	$r_{3}$	$r_{4}$	$r_{5}$	$r_{6}$
$v_{1}$	**1**	1	0	**1**	1	0	$v_{1}$	**1**	1	0	**1**	1	**0**	$v_{1}$	1	**1**	**0**	**1**	**1**	**0**
$v_{2}$	0	0	1	0	0	1	$v_{2}$	**0**	0	1	**0**	0	**1**	$v_{2}$	0	**0**	**1**	**0**	**0**	**1**
$v_{3}$	0	1	0	0	1	0	$v_{3}$	0	1	0	0	1	0	$v_{3}$	0	**1**	**0**	**0**	**1**	**0**
$v_{4}$	**1**	0	0	**0**	0	0	$v_{4}$	**1**	0	0	**0**	0	**0**	$v_{4}$	1	0	0	0	0	0
$v_{5}$	**0**	0	0	**1**	0	0	$v_{5}$	**0**	0	0	**1**	0	**0**	$v_{5}$	0	**0**	**0**	**1**	**0**	**0**
$v_{6}$	0	1	1	0	0	0	$v_{6}$	0	1	1	0	0	0	$v_{6}$	0	**1**	**1**	**0**	**0**	**0**
$v_{7}$	**0**	0	0	**0**	1	1	$v_{7}$	**0**	0	0	**0**	1	**1**	$v_{7}$	0	**0**	**0**	**0**	**1**	**1**

**Table 5:**
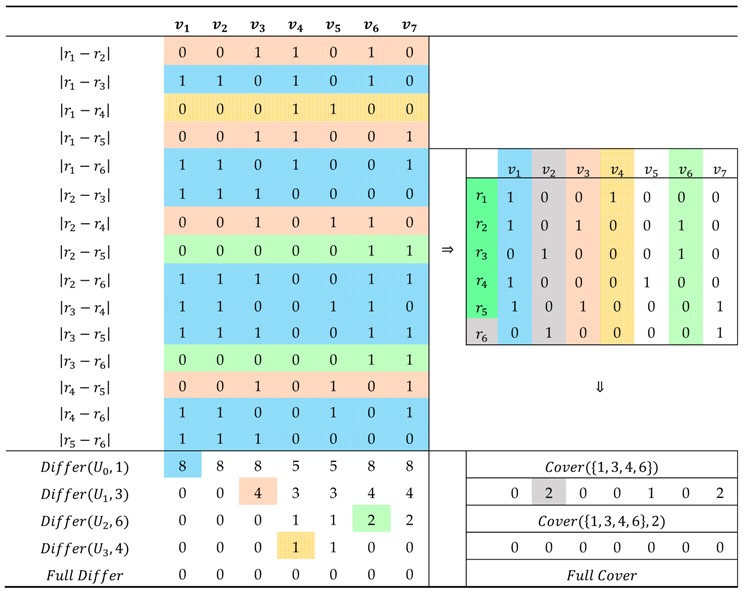
The process of greedy algorithm considering ‘differentiating first’ principle.

**Table 6:** The process of replacing scanning with counting in Figure 1 example.

N_re = 1		3, 4, 6, 2	1, 3, 4, 6	1, 4, 6, 2	1, 3, 6, 2	1, 3, 4, 2					
Full-rank?	$H_{s}$	1	1	0	0	0					
	$H_{c}$	1	1	---	---	---					
Combination	$U_{s}$	**3, 4, 6, 2**	**1, 3, 4, 6**								
	$U_{c}$	**1**	**2**								
N_re = 2		4, 6, 2	3, 6, 2	3, 4, 6	3, 4, 2	1, 3, 4	1, 4, 6	1, 3, 6	1, 3, 4	1, 6, 2	1, 4, 2
Full-rank?	$H_{s}$	1	1	1	0	0	0	0	0	0	0
	$H_{c}$	1	1	1	---	---	---	---	---	---	---
Combination	$U_{s}$	**4, 6, 2**	**3, 6, 2**	**3, 4, 6**							
	$U_{c}$	**1, 3**	**1, 4**	**1, 2**							
N_re = 3		3, 6	2, 6	1, 3	1, 4	1, 6	1, 2	3, 4	3, 2	4, 6	4, 2
Full-rank?	$H_{s}$	1	1	0	0	0	0	0	0	0	0
	$H_{c}$	1	1	---	---	---	---	---	---	---	---
Combination	$U_{s}$	**3, 6**	**2, 6**								
	$U_{c}$	**1, 2, 4**	**1, 3, 4**								

**Table 7:** The O-D pairs and links relationship in Nguyen-Dupuis network.

O-D	Route	Links						OD	Route	Links					
1–2	1	1	11	14	18	20		3–1	26	6	38	24	9		
1–2	2	2	35	14	18	20		3–4	27	5	33	28	23		
1–2	3	2	36	20				3–4	28	6	38	23			
1–3	4	1	11	14	19	31		3–12	29	5	32	17	16		
1–3	5	1	11	15	29	31		3–12	30	5	33	27	16		
1–3	6	1	12	25	29	31		4–2	31	7	11	14	18	20	
1–3	7	1	12	26	37			4–2	32	8	25	29	30		
1–3	8	2	35	14	19	31		4–2	33	8	25	29	32	18	20
1–3	9	2	35	15	29	31		4–3	34	8	25	29	31		
1–8	10	1	11	14	18			4–3	35	8	26	37			
1–8	11	2	35	14	18			4–8	36	7	11	14	18		
1–8	12	2	36					4–8	37	8	25	29	32	18	
2–1	13	3	21	17	13	9		8–1	38	21	17	13	9		
2–1	14	3	21	17	16	34		8–1	39	21	17	16	34		
2–1	15	3	22	34				8–1	40	22	34				
2–4	16	3	21	17	13	10		8–4	41	21	17	13	10		
2–4	17	3	21	19	33	28	23	8–4	42	21	19	33	28	23	
2–4	18	4	33	28	23			8–12	43	21	17	16			
2–12	19	3	21	17	16			8–12	44	22					
2–12	20	3	22					12–2	45	35	14	18	20		
3–1	21	5	32	17	13	9		12–2	46	36	20				
3–1	22	5	32	17	16	34		12–3	47	35	14	19	31		
3–1	23	5	33	27	13	9		12–3	48	35	15	29	31		
3–1	24	5	33	27	16	34		12–8	49	35	14	18			
3–1	25	5	33	28	24	9		12–8	50	36					

**Table 8:**
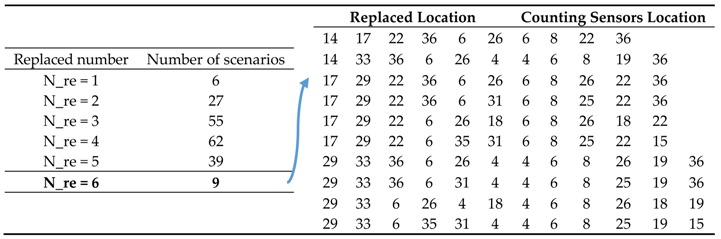
Combination deployment schemes for the Nguyen-Dupuis network.
